# Can nitrocobalamin be reduced by ascorbic acid to nitroxylcobalamin? Some surprising mechanistic findings

**DOI:** 10.1007/s00775-018-1540-1

**Published:** 2018-02-12

**Authors:** Justyna Polaczek, Łukasz Orzeł, Grażyna Stochel, Rudi van Eldik

**Affiliations:** 1 Faculty of Chemistry, Jagiellonian University, Gronostajowa 2, 30-387 Kraków, Poland; 2 Department of Chemistry and Pharmacy, University of Erlangen-Nuremberg, Egerlandstrasse 1, 91058 Erlangen, Germany

**Keywords:** Nitrocobalamin, Nitroxylcobalamin, Nitrite, Ascorbic acid, Redox reactions

## Abstract

**Abstract:**

Despite detailed studies on nitroxylcobalamin (CblNO) formation, the possible intracellular generation of CblNO via reduction of nitrocobalamin (CblNO_2_) remains questionable. To study this further, spectroscopic studies on the reaction of CblNO_2_ with the intracellular antioxidant ascorbic acid (HAsc^−^) were performed in aqueous solution at pH < 5.0. It was found that nitroxylcobalamin is the final product of this interaction, which is not just a simple reaction but a rather complex chemical process. We clearly show that an excess of nitrite suppresses the formation of CblNO, from which it follows that ascorbic acid cannot reduce coordinated nitrite. We propose that under the influence of ascorbic acid, nitrocobalamin is reduced to Cbl(II) and nitric oxide (^·^NO), which can subsequently react rapidly to form CblNO. It was further shown that this system requires anaerobic conditions as a result of the rapid oxidation of both Cbl(II) and CblNO.

**Graphical Abstract:**

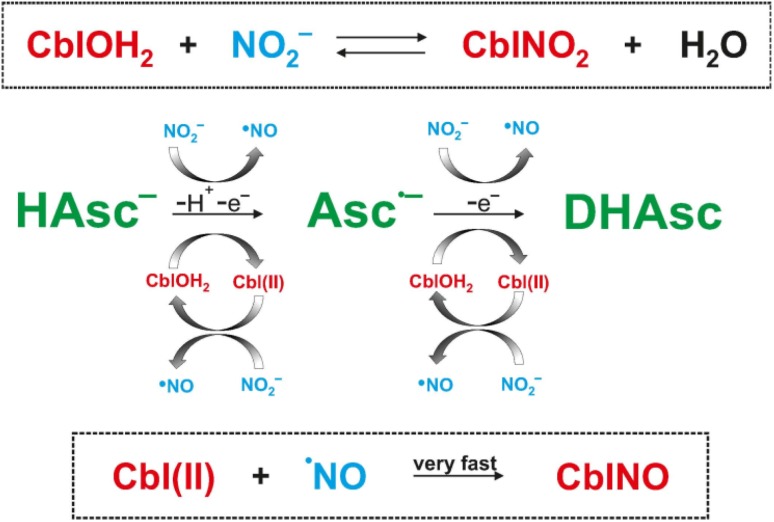

**Electronic supplementary material:**

The online version of this article (10.1007/s00775-018-1540-1) contains supplementary material, which is available to authorized users.

## Introduction

Nitroxylcobalamin (CblNO, formally Co^III^–NO^−^) [1] is one of the most interesting forms of Vitamin B_12_ that was shown to be stable in biological systems [2, 3]. Under physiological conditions, CblNO can be produced in a very efficient reaction between the major intracellular form of Vitamin B_12r_, viz. cob(II)alamin [4], and nitric oxide (^·^NO) for which *k* = 7.4 × 10^8^ M^−1^ s^−1^ and *K*_NO_ ≈ 1 × 10^8^ M^−1^ at 25 °C [5–7]. It has been postulated that cobalamins show the potential to eliminate excess ^·^NO from organisms [8, 9] since the Co^III^–NO^−^ complex can be protonated at neutral pH to form Co^III^–NOH, which in turn can undergo aquation to release HNO. The latter species is known to dimerize and decompose to water and gaseous N_2_O in aqueous solution [10].

**Scheme 1:**
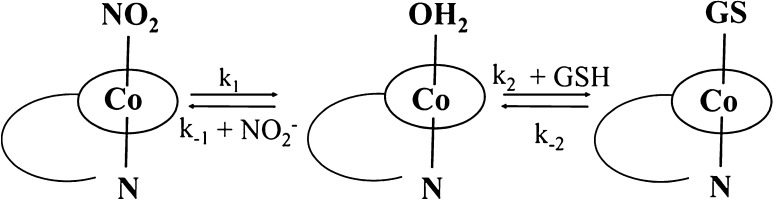
Schematic presentation of the reaction between CblNO_2_ and GSH

CblNO is extremely air sensitive [6, 11, 12] and in the presence of oxygen it rapidly oxidizes to nitrocobalamin (CblNO_2_) [6, 11–13]. However, the oxidation of CblNO is not just a simple reaction [14]. According to Brasch et al., the mechanism of CblNO oxidation under certain conditions is rather complex, with multiple products that can be formed [15]. Oxidation of CblNO is a reversible process in which CblNO_2_ can be reduced by strong reducing agents to reform CblNO. Brasch et al. studied the reaction between CblNO_2_ and one of the strongest intracellular antioxidants glutathione (GSH), as one of the possibilities to form CblNO [16]. An important conclusion from this work is that for a pH between 4 and 7, reduction of CblNO_2_ to CblNO was not observed! Furthermore, the main product of this reaction is glutathionylcobalamin (CblGS). The reported kinetic data suggest that the observed reaction is a two-step process which involves aquacobalamin (CblOH_2_) as an intermediate that rapidly reacts with GSH to form CblGS (Scheme 1) [16].

**Scheme 2:**
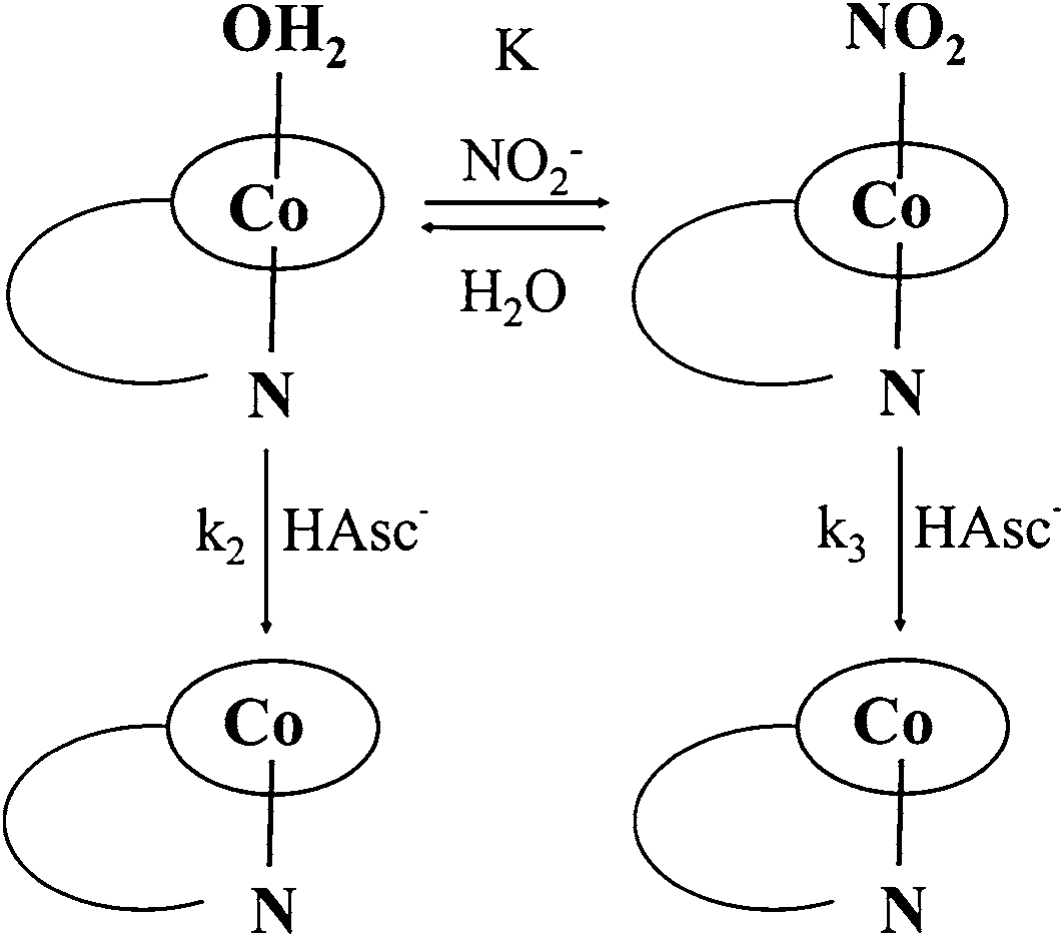
Suggested mechanism for the reaction between CblNO_2_ and HAsc^−^ at pH ~ 7

Our recent study [17] showed that at neutral pH (pH 7.2, 0.1 M Tris buffer, 25 °C), CblNO_2_ can react with another biological reducing agent, ascorbic acid (HAsc^−^), to form reduced cobalamin (Cbl(II)) as product, but not CblNO as was hoped. Kinetic data showed that both CblNO_2_ and CblOH_2_ present in an equilibrium mixture react with HAsc^−^, but CblOH_2_ reacts ca. two orders of magnitude faster than CblNO_2_ (Scheme 2), i.e. *k*_2_ ≫ *k*_3_. Thus, on addition of ascorbic acid to the equilibrium mixture of CblOH_2_ and CblNO_2_, it reacts rapidly with CblOH_2_ which in turn is reformed through the aquation of CblNO_2_ (*k*_1_ = 1.4 × 10^−2^ s^−1^ at 25 °C) and so represents the major reactive species in solution. Furthermore, in the presence of a large excess of nitrite the reduction of CblOH_2_ can be drastically inhibited due to the formation of CblNO_2_ [17]. Recent work in our laboratories indicated that this system shows a more interesting behavior under slightly different experimental conditions.

## Experimental section

### Materials

Hydroxocobalamin hydrochloride (HOCbl·HCl, ≥ 98%) was purchased from Sigma-Aldrich, sodium nitrite was purchased from LPPH and ascorbic acid was obtained from Polfa Kraków. Acetic acid (CH_3_COOH,  $\ge$ 99.5–99.9%) and sodium hydroxide (NaOH, $\ge$ 98.8%) were obtained from a range of suppliers (Sigma-Aldrich, Merck, Fisher Scientific or POCH). All chemicals used throughout this study were of analytical grade or better.

### General methods

All solutions were prepared in de-ionized water using a water purification system. Strictly anaerobic solutions were prepared using appropriate air-free techniques and handling the solutions in appropriate glassware. Oxygen-free argon or nitrogen was used to deoxygenate the reactant solutions. UV–Vis spectral measurements were carried out in screw-cap cuvettes equipped with a silicone septum. pH measurements were carried out at room temperature using a HI 221 (Hanna Instruments) pH-meter equipped with an AmpHel glass electrode filled with a 3 M KCl solution.

### UV–Vis spectroscopy

UV–Vis spectra and kinetic data were recorded on Perkin Elmer Lambda 25 spectrophotometer equipped with a thermostated (25.0 ± 0.1 °C) cell holder (Perkin Elmer PTP-6 Peltier System). All data were analyzed using Origin Lab software.

## Results and discussion

**Fig. 1:**
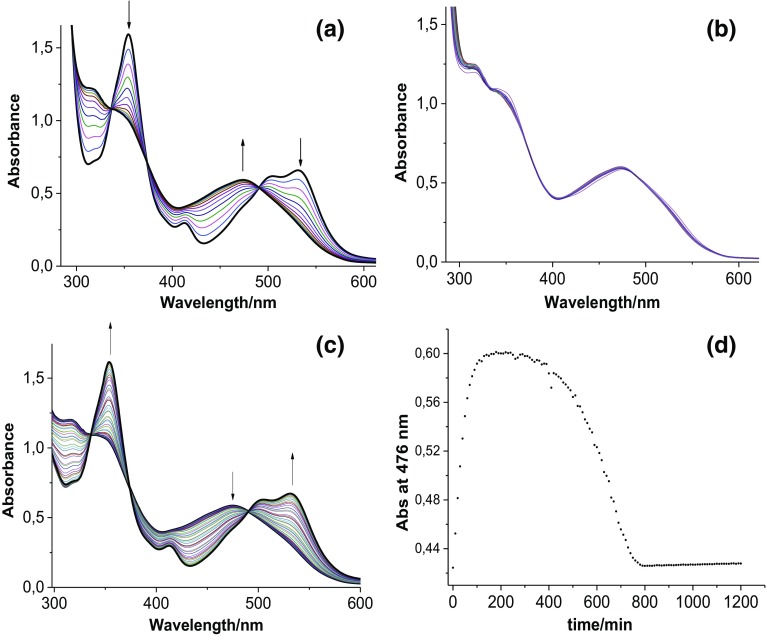
**a** Spectral changes observed for the reaction between CblNO_2_ (8.6 × 10^−5^ M, obtained by mixing CblOH_2_ and NO_2_^−^, [NO_2_^−^]/[CblOH_2_] = 5) and HAsc^−^ (8.6 × 10^−4^ M) at pH 4.3 (0.1 M acetate buffer, 25 °C, Ar atmosphere) during the first 100 min, **b** between 100 and 400 min, **c** between 400 and 1200 min from the start of the reaction. Spectra were recorded every 1 min. **d** Plot of absorbance at 476 nm vs. time

After completion of our studies at pH ~ 7, we decided to check the influence of ascorbic acid on the reaction with CblNO_2_ at a more acidic pH ≤ 5, where ascorbic acid is mainly present in the mono-protonated form HAsc^−^ and the much weaker reducing agent H_2_Asc, depending on the selected pH (p*K*_a1_ = 4.1 and p*K*_a2_ = 11.3 [18]). Under these conditions, mixtures of H_2_Asc and HAsc^−^ have significantly weaker reducing properties than at pH ~ 7, where ascorbic acid is mainly present as HAsc^−^ with traces of the stronger reducing agent Asc^2−^ [18–23]. UV–Vis spectra recorded during the reaction between CblNO_2_ and HAsc^−^ at pH < 5 (0.1 M acetate buffer, 25 °C, Ar atmosphere) indicated that CblNO_2_ (*λ*_max_ = 354, 413 and 532 nm) is not converted to Cbl(II) as before [17], but appears to be converted directly to nitoxylcobalamin, CblNO (*λ*_max_ = 316, 344 and 475 nm) [6] with isosbestic points at 335, 373, 490 nm (Fig. 1a). Under conditions of pH 4.3 (0.1 M acetate buffer, 25 °C, Ar atmosphere), [CblNO_2_] = 8.6 × 10^−5^ M (obtained by mixing CblOH_2_ and NO_2_^−^, [NO_2_^−^]/[CblOH_2_] = 5) and [HAsc^−^] = 8.6 × 10^−4^ M, CblNO was formed within 100 min from the start of the reaction. During the next 300 min practically no spectral changes were observed, from which we conclude that CblNO is stable in solution under the selected conditions (Fig. 1b). However, during the next 400 min the absorbance at 476 nm decreased to the initial value and the UV–Vis spectra clearly showed that CblNO_2_ was reformed fully (isosbestic points at 335, 373, 490 nm) (Fig. 1c, d).

**Fig. 2:**
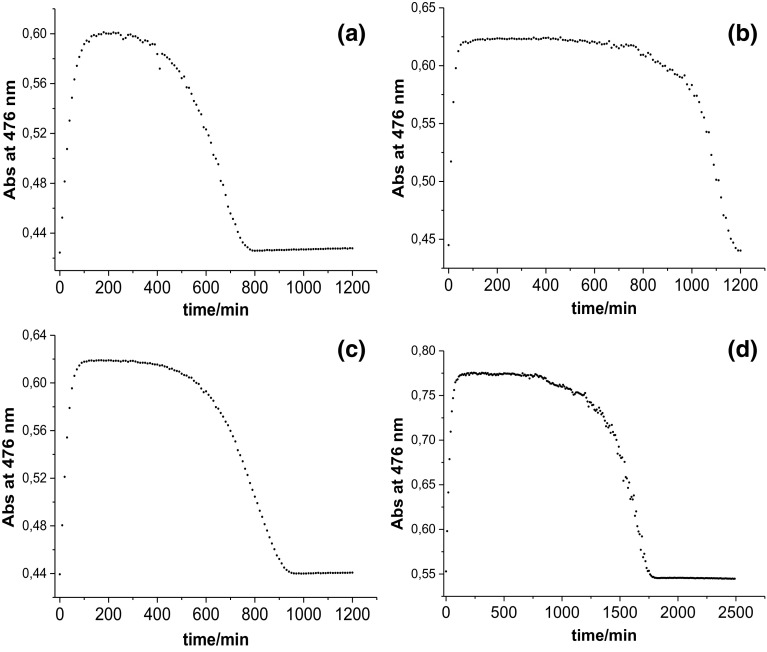
Plot of absorbance at 476 nm vs. time for the reaction between CblNO_2_ (8.6 × 10^−5^ M, obtained by mixing CblOH_2_ with NO_2_^−^) and HAsc^−^ at pH 4.3 (0.1 M acetate buffer, 25 °C, Ar atmosphere). Concentration ratio for [CblOH_2_]:[NO_2_^−^]:[HAsc^−^]: **a** 1:5:10, **b** 1:5:20, **c** 1:10:20 and **d** 1:10:30

Further studies showed that CblNO was formed faster and remained for longer times in the reaction mixture when higher concentrations of HAsc^−^ were used (compare Fig. 2a, b and d), whereas increasing the nitrite concentration slowed down the conversion of CblNO_2_ to CblNO (compare Fig. 2b and c). In a more detailed series of experiments, the nitrite concentration was varied over a wider concentration range while keeping the ascorbate concentration constant. The results reported in Figure S1 (Supporting Information) show that the initial phase of the reaction indeed slows down significantly on increasing the nitrite concentration. The kinetic data could be fitted best with a zero-order process for the initial changes in absorbance with time, and the slopes of such plots as a function of CblOH_2_ concentration show typical saturation kinetics (see Figure S1e). From the value of *K* = 1 × 10^5^ M^−1^ (see Scheme 2) reported at 25 °C [17], it can be estimated that the concentration of CblOH_2_ in solution decreases from 1.95 × 10^−6^ to 4.97 × 10^−7^ M on increasing the nitrite concentration from 4.3 × 10^−4^ to 1.72 × 10^−3^ M for the experiments reported in Figure S1. This clearly demonstrates the effect of the nitrite concentration on the rate of formation of CblNO. In the presence of a large excess of nitrite, no reaction was observed at all, suggesting that nitrite completely suppresses the formation of CblNO.

**Fig. 3:**
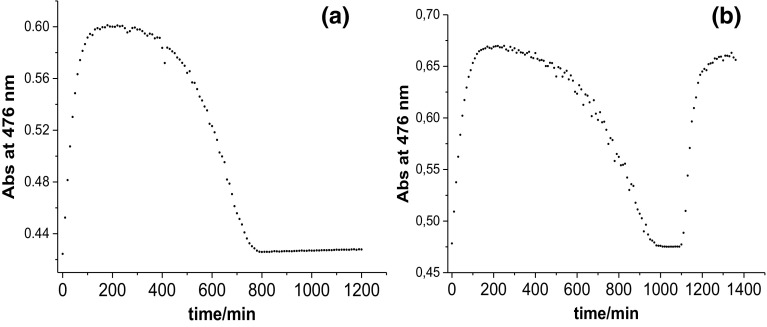
**a** Plot of absorbance at 476 nm vs. time for the reaction between CblNO_2_ and HAsc^−^. **b** Plot of absorbance at 476 nm vs. time for the reaction between CblNO_2_ and HAsc^−^ with the addition of extra HAsc^−^ after 1100 min from the start of the reaction

Additional studies were performed to check whether after almost 20 h from the start of the reaction, it is possible to reduce the final CblNO_2_ product by the addition of an extra amount of ascorbic acid. Surprisingly, addition of an extra portion of HAsc^−^ resulted once again in the formation of CblNO as shown in Fig. 3b by the increase in absorbance at 476 nm from 1100 to 1400 min.

A blank experiment was performed in the absence of cobalamin in which solutions of nitrite and HAsc^−^ were mixed under exactly the same conditions as we used in the experiments with cobalamin. The absorbance maximum at 262 nm which comes from HAsc^−^ decreases with time to reach the pre-reaction value after 20 h from the start of the reaction (Figure S2, Supporting Information). We ascribe these findings to slow side reactions between ascorbic acid and nitrite, and/or slow diffusion of oxygen into the sealed cuvettes over longer periods of time (see further discussion).

**Fig. 4:**
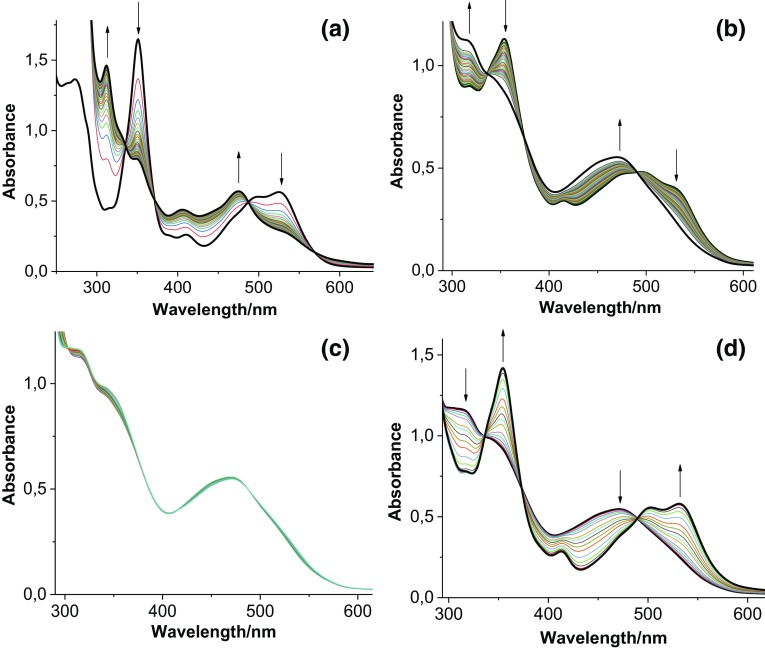
Spectral changes observed for the reaction between CblOH_2_ (8.6 × 10^−5^ M) and HAsc^−^ (8.6 × 10^−4^ M) at pH 4.3 (0.1 M acetate buffer, 25 °C, Ar atmosphere: **a**), and for the reaction of Cbl(II) obtained in **a** with NO_2_^−^ (4.3 × 10^−4^ M; **b**, **c** and **d**) between 0 and 60 min (**b**), between 60 and 600 min (**c**) and between 600 and 1200 min from the start of the reaction (**d**). In **a** and **b** spectra are recorded every 1 min, whereas in **c** and **d**  every 30 min

In addition, we could show that by changing the sequence of mixing the reagents this resulted in totally different spectral changes. On mixing first CblOH_2_ with HAsc^−^ (before addition of nitrite), CblOH_2_ was fully reduced to Cbl(II) as shown in Fig. 4a. Subsequent addition of nitrite led to the rapid formation of CblNO_2_ and not CblNO, i.e. Cbl(II) is oxidized to CblOH_2_ which reacts rapidly with nitrite to form CblNO_2_ followed by its reduction to CblNO (Fig. 4b). The CblNO complex remains stable for some time (Fig. 4c) before it slowly converts back to CblNO_2_ due to the depletion of ascorbate (Fig. 4d).

We also used Fe^II^(EDTA) as a very efficient trap for the intermediate formation of ^·^NO [6, 24]. On mixing typical concentrations of nitrite and ascorbate under Ar atmosphere and allowing them to react for 2 h, the addition of Fe^II^(edta) immediately resulted in the formation of Fe(edta)NO as shown in Figure S3 (Supporting Information). This is clear evidence for the intermediate formation of ^·^NO during the reduction of nitrite by ascorbate under the selected conditions of this study.

The results reported above show that it is indeed possible to observe the formation of CblNO under milder reducing conditions with ascorbic acid at pH < 5. In terms of the biological relevance of these findings, we repeated a series of measurements where the pH was systematically decreased from 7.2 to 5.0, to see where the changeover from Cbl(II) to CblNO as reaction product occurs. In these experiments a typical concentration ratio of [CblOH_2_]:[NO_2_^−^]:[HAsc^−^] = 1:5:10 was selected as done in Fig. 2. On decreasing the pH the reaction product changed from only Cbl(II) (pH 7.2, Figure S4, Supporting Information) to a mixture of Cbl(II) and CblNO (pH 5.5, Figure S5, Supporting Information), to only CblNO (pH 5.0, Figure S6, Supporting Information). It follows that as we go to milder reducing conditions by lowering the pH, only Cbl(NO) is formed at pH ≤ 5.0, which must be related to the pH dependence of the redox potential for the two-electron ascorbic acid/dehydroascorbate transformation. According to the Pourbaix diagram for this transformation [18], the redox potential of H_2_Asc at pH 0 is + 0.4 V, of H_2_Asc/HAsc^−^ at pH 4.1 (p*K*_a1_) is + 0.16 V, for HAsc^−^ at pH 7.0 (8.0) is + 0.07 (+ 0.04 V), and for HAsc^−^/Asc^2−^ at pH 11.3 (p*K*_a2_) is − 0.15 V. These data clearly show the large change in redox potential to a significantly stronger reducing agent on increasing the pH of the solution.

The challenge now will be to find a biologically relevant reducing agent that under mild reaction conditions will reduce CblNO_2_ to CblNO at pH 7.4.

### Mechanistic interpretation

The results of this study have clearly demonstrated that it is possible to obtain stable solutions of CblNO in the presence of a reducing agent starting from CblNO_2_ under well-selected reaction conditions. The remaining question is how can we account for the different reaction steps observed?

Our initial idea was that coordinated nitrite can be reduced by ascorbic acid to form the nitroxyl complex CblNO, since this is what the observed spectral changes tell us, thus a direct reaction from CblNO_2_ to CblNO. However, in these experiments performed at a pH < 5 we noticed that the conversion of CblNO_2_ to CblNO slowed down on increasing the nitrite concentration. In the presence of a large excess of nitrite, no reaction was observed at all. Thus, nitrite suppresses the formation of CblNO, from which we can conclude that ascorbic acid cannot reduce coordinated nitrite. This means that the remaining low concentration of CblOH_2_ in solution is reduced by the added HAsc^−^ as found in our earlier report [17] and presented in reactions (1) and (2), where DHAsc represents dehydroascorbate.

1 $${\text{CblOH}}_{2} + {\text{ HAsc}}^{ - } \to {\text{Cbl}}\left( {\text{II}} \right) \, + {\text{ Asc}}^{ \cdot - }$$

2 $${\text{CblOH}}_{2} + {\text{ Asc}}^{ \cdot - } \to {\text{Cbl}}\left( {\text{II}} \right) \, + {\text{ DHAsc}}$$

In a subsequent reaction, Cbl(II) can be oxidized by nitrite to form CblOH_2_ and ^·^NO.

3 $${\text{Cbl}}\left( {\text{II}} \right) \, + {\text{ NO}}_{ 2}^{ - } \to {\text{CblOH}}_{2} + {}^{ \cdot }{\text{NO}}$$

4 $${\text{Cbl}}\left( {\text{II}} \right) \, + {}^{ \cdot }{\text{NO }} \to {\text{CblNO}}$$

The CblOH_2_ formed in reaction (3) is immediately reduced by HAsc^−^ in reactions (1) and (2) and the nitroxyl product is formed in the very fast radical coupling of Cbl(II) and ^·^NO (*k* = 7.4 x 10^8^ M^−1^ s^−1^ [6]), reaction (4). Thus, reactions (1) to (4) account for the observation that CblNO was formed faster and remained for longer times in the reaction mixture when higher concentrations of HAsc^−^ were used, whereas increasing nitrite concentration slowed down the conversion of CblNO_2_ to CblNO. At the point where ascorbic acid is depleted, CblNO can react with excess ^·^NO to yield CblOH_2_ and N_2_O, similar to that found for the reaction between free HNO and ^·^NO to form NO_2_^−^ and N_2_O [25]. Subsequently, CblOH_2_ is converted to CblNO_2_, the final reaction product in the presence of excess nitrite.

There are some side reactions (5) and (6) that can contribute to the formation of ^·^NO on a longer timescale and add to the redox cycling that causes the overall decomposition of ascorbic acid to DHAsc and the reformation of CblNO_2_. These reactions have been known for almost 60 years [26].

5 $${\text{NO}}_{2}^{ - } + {\text{ HAsc}}^{ - \cdot } \to {}^{ \cdot }{\text{NO }} + {\text{ Asc}}^{ \cdot - }$$

6 $${\text{NO}}_{2}^{ - } + {\text{ Asc}}^{ \cdot - } \to {}^{ \cdot }{\text{NO }} + {\text{ DHAsc}}$$

In this case, the produced ^·^NO will react rapidly with Cbl(II) to form CblNO in reaction (4).

In addition, traces of oxygen can oxidize ^·^NO to ^·^NO_2_ and reform nitrite according to reactions (7)–(9).

7 $$2{}^{ \cdot }{\text{NO }} + {\text{ O}}_{2} \to 2{}^{ \cdot }{\text{NO}}_{2}$$

8 $$^{ \cdot } {\text{NO }} + {}^{ \cdot }{\text{NO}}_{2} \to {\text{N}}_{2} {\text{O}}_{3}$$

9 $${\text{N}}_{2} {\text{O}}_{3} + {\text{ H}}_{2} {\text{O}} \to 2{\text{HONO}} \rightleftarrows 2{\text{H}}^{ + } + \, 2{\text{NO}}_{2}^{ - }$$

Reactions (5)–(9) can account for the reformation of nitrite and CblNO_2_, as well as the overall depletion of ascorbic acid from the solution over longer reaction times.

Traces of oxygen can also oxidize Cbl(II) to form CblOH_2_, which in turn will be reduced by HAsc^−^ back to Cbl(II) [27–29], by which more ascorbic acid will be used. But at the same time, oxygen will be reduced to form superoxide, peroxide and OH^·^, which are stronger oxidants and can account for more redox cycling to occur during which ascorbic acid will be depleted, reactions (10)–(12). Finally, CblNO can also react with dioxygen to form CblNO_2_ and CblOH_2_ in a complex process involving OH^·^ and ^·^NO_2_ intermediates [15].

10 $${\text{Cbl}}\left( {\text{II}} \right) \, + {\text{ O}}_{2} \to {\text{CblOH}}_{2} + {\text{ O}}_{2}^{ - }$$

11 $${\text{Cbl}}\left( {\text{II}} \right) \, + {\text{ O}}_{2}^{ - } \to {\text{CblOH}}_{2} + {\text{ O}}_{2}^{2 - }$$

12 $${\text{O}}_{2}^{2 - } + \, {\text{ H}}^{ + } \rightleftarrows {\text{HO}}_{2}^{ - } + {\text{ H}}^{ + } \rightleftarrows{\text{H}}_{2} {\text{O}}_{2} \rightarrow 2{\text{OH}}^{ \cdot }$$

**Scheme 3:**
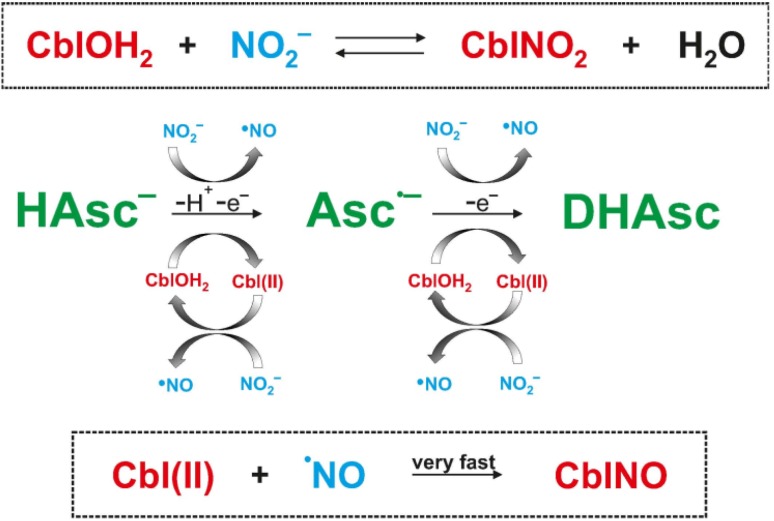
Overall reaction sequence for the redox cycling of CblNO_2_, CblNO and CblOH_2_ in the presence of ascorbate

The overall reaction sequence for the redox cycling of CblNO_2_, CblNO and CblOH_2_ in the presence of ascorbate is presented in Scheme 3. The main redox cycling components are marked in red and blue, whereas the oxidation of ascorbate in green. Complications caused by traces of oxygen that could not be avoided over very long reaction times are not included in the scheme for clarity reasons, but are given in reactions (7)–(12).

The suggested reaction sequence in Scheme 3 is based on a one-electron reduction process by which nitrite is reduced to ^·^NO by ascorbate. From the recent literature [30, 31] it is known that free ^·^NO can be reduced by ascorbic acid to form HNO, which in turn can react with CblOH_2_ to form CblNO. Brasch and coworkers [32] demonstrated that using Angeli’s salt (HN_2_O_3_^−^) as source of HNO, the formation of CblNO can occur at pH > 10.8, where the rate-determining step is the release of HNO by Angeli’s salt, such that no mechanistic details about the mechanism of the reaction between CblOH_2_/CblOH and HNO/NO^−^ could be revealed. At present, it is questionable whether further reduction of ^·^NO to HNO and a direct reaction of CblOH_2_ with HNO to form CblNO can account for the results presented in this study.

All in all, our goal to find suitable reaction conditions to produce CblNO from CblNO_2_ in the presence of a reducing agent over a long period of time was successful and has added to the overall understanding of the complex reaction system.

## Conclusions

The reaction of CblNO_2_, one of the naturally occurring forms of cobalamin, with ascorbate has been studied by UV–Vis spectroscopy. The present study provides mechanistic information on this reaction at pH < 5. Under this condition, the only product of the reaction is CblNO. However, for the reduction of CblNO_2_ by ascorbate, no direct evidence for the reduction of coordinated nitrite could be found. On the contrary, we showed that excess of nitrite suppressed the formation of CblNO, from which we can conclude that ascorbic acid/ascorbate cannot reduce coordinated nitrite. We suggest that the studied system is not just a simple reaction, but a rather complex chemical process. During the reaction of ascorbic acid with nitrocobalamin, the first products formed are the reduced form of Vitamin B_12_ (Cbl(II)) and nitric oxide (^·^NO) that subsequently react rapidly to form CblNO. Our results show that the studied reactions are extremely oxygen sensitive due to the reverse oxidation of both Cbl(II) and CblNO to CblOH_2_ and CblNO_2_, respectively.

## Electronic supplementary material

Below is the link to the electronic supplementary material.

Supplementary material 1 (PDF 420 kb)
