# Supplementary material for: Can nitrocobalamin be reduced by ascorbic acid to nitroxylcobalamin? Some surprising mechanistic findings
Source: J Biol Inorg Chem. 2018 Feb 12;23(3):377–83. doi: 10.1007/s00775-018-1540-1 (PMC5940710; doi:10.1007/s00775-018-1540-1)
Supplement: Supplementary file 1 — Supplementary material 1 (PDF 420 kb) [file 775_2018_1540_MOESM1_ESM.pdf]

Supporting Information

Can nitrocobalamin be reduced by ascorbic acid to nitroxylcobalamin? Some surprising mechanistic findings

Justyna Polaczek,[a] Łukasz Orzeł,[a] Grażyna Stochel[a] and Rudi van Eldik[a,b,]*

[a] Faculty of Chemistry, Jagiellonian University, Gronostajowa 2, 30-387 Kraków, Poland.

[b] Department of Chemistry and Pharmacy, University of Erlangen-Nuremberg, Egerlandstrasse 1, 91058 Erlangen, Germany. E-mail: rudi.vaneldik@fau.de

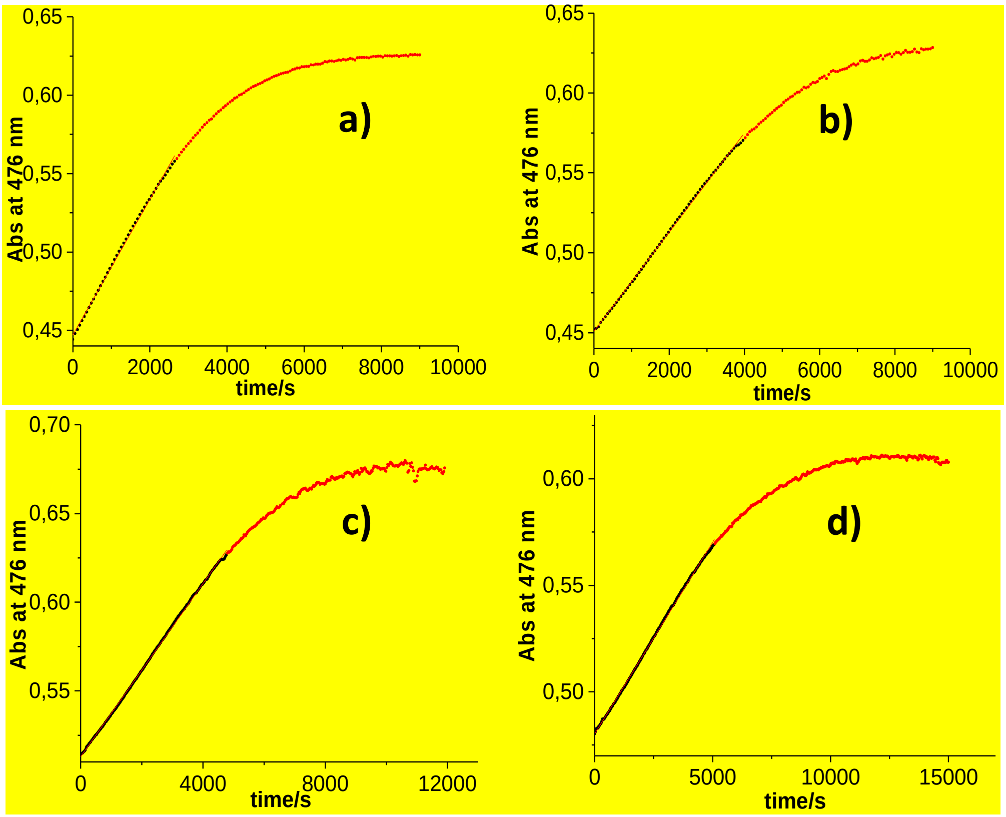

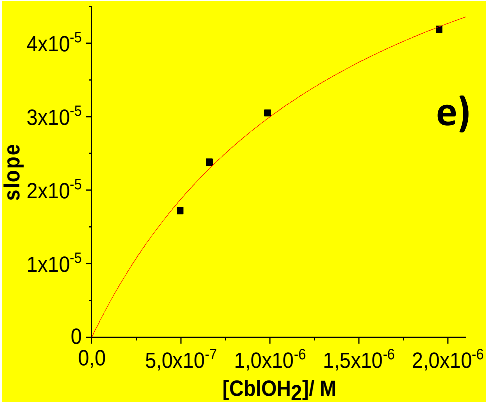

Figure S1. Typical kinetic traces recorded for the formation of CblNO as a function of nitrite concentration. Experimental conditions: $[CblOH_2]$ = 8.6 x 10^{-5}; $[HAsc^-]$ = (1.7 x 10^{-3} M) at pH = 4.3 (0.1 M acetate buffer, 25 °C, Ar atmosphere); $[NO_2^-]$ = 4.3 x 10^{-4} (a), 8.6 x 10^{-4} (b), 12.9 x 10^{-4} (c) and 17.2 x 10^{-4} (d) M. (e) Plot of initial slope versus $[CblOH_2]$ fitted with the rate law: Rate = $kK[CblOH_2]/\{1 + K[CblOH_2]\}$, typical for saturation kinetics.

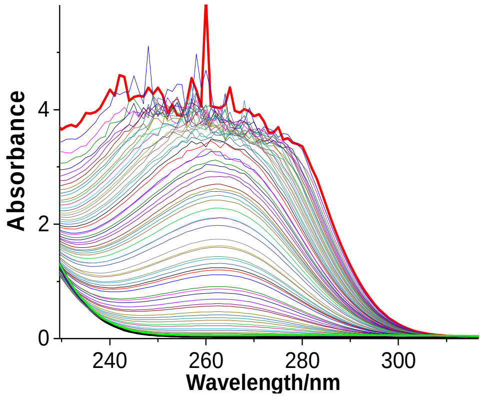

Figure S2. Spectral changes observed for the reaction between NO_2^- (4.3 x 10^{-4} M) and $HAsc^-$ (8.6 x 10^{-4} M) at pH = 4.3 (0.1 M acetate buffer, 25 °C, Ar atmosphere) during 1200 min from the start of the reaction. Spectra were recorded every 1 min.

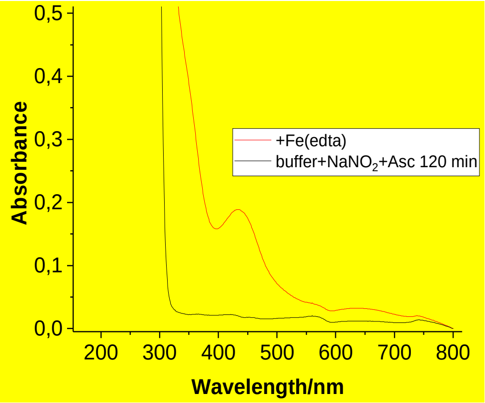

Figure S3. Evidence for the intermediate formation of $^{\bullet}NO$ produced during the reaction of nitrite with ascorbate, tested with Fe^{II}(edta) as trapping reagent. The intense band at 435 nm is characteristic for the formation of Fe(edta)NO. Experimental conditions: $[NO_2^-] = 4.3 \times 10^{-4}$ M; $[HAsc^-] = 8.6 \times 10^{-4}$ M; pH = 4.3 (0.1 M acetate buffer, 25 °C, Ar atmosphere); reaction time 2 h prior to addition of 1 mM Fe^{II}(edta).

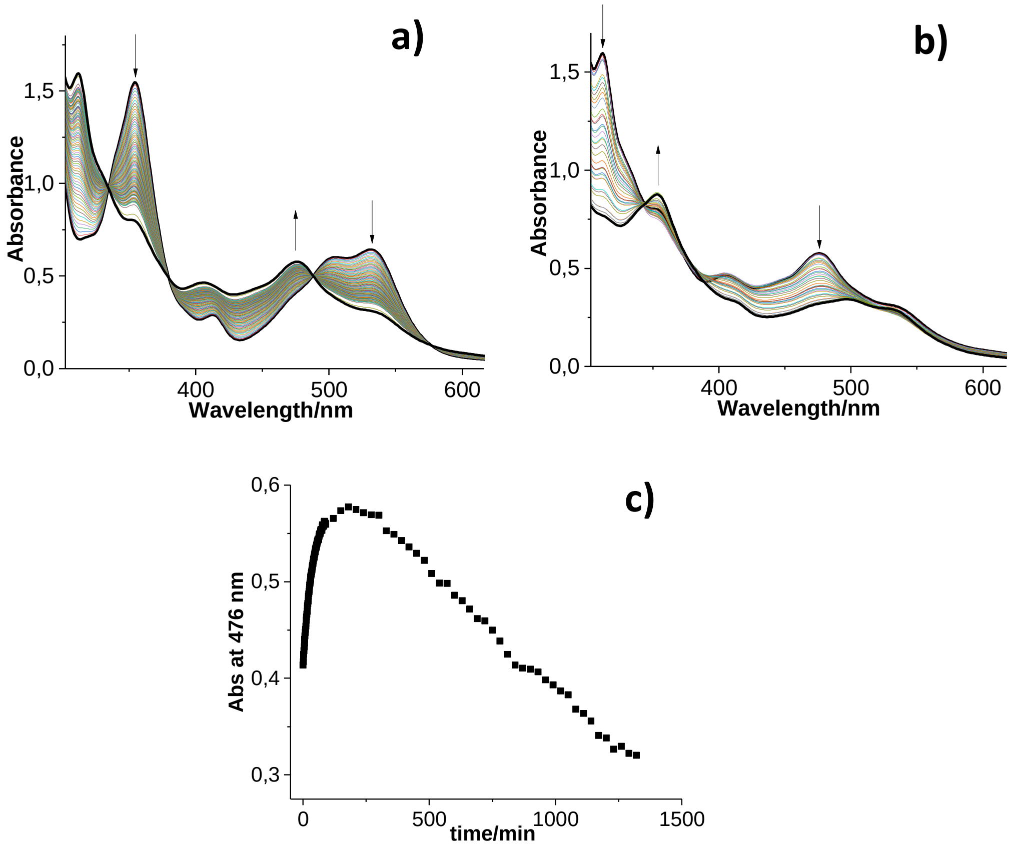

Figure S4. a) Spectral changes observed for the reaction between $CblNO_2$ (8.6×10^{-5} M, obtained by mixing $CblOH_2$ and NO_2^-, $[NO_2^-]/[CblOH_2] = 5$) and $HAsc^-$ (8.6×10^{-4} M) at pH = 7.2 (0.1 M Tris buffer, 25 °C, Ar atmosphere) during the first 180 min, b) between 180 and 1300 min from the start of the reaction. Spectra were recorded every 1 min. c) Plot of absorbance at 476 nm vs. time.

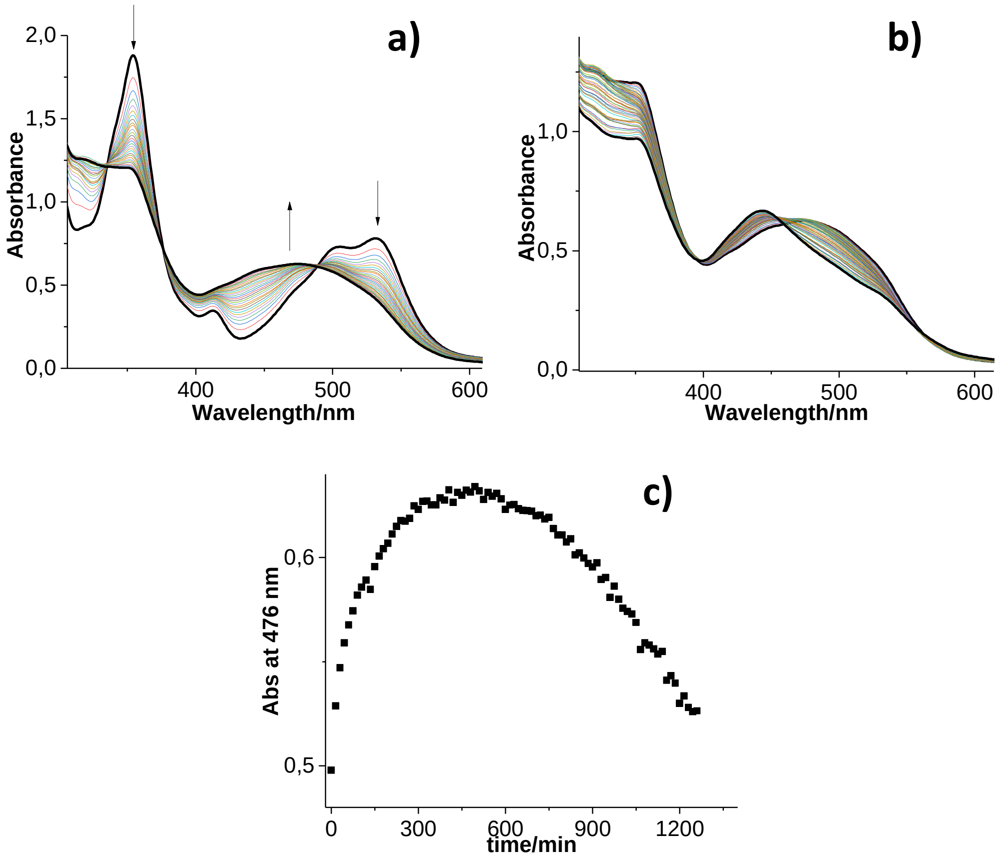

Figure S5. a) Spectral changes observed for the reaction between $CblNO_2$ (8.6×10^{-5} M, obtained by mixing $CblOH_2$ and NO_2^-, $[NO_2^-]/[CblOH_2] = 5$) and $HAsc^-$ (8.6×10^{-4} M) at pH = 5.5 (0.1 M acetate buffer, 25 °C, Ar atmosphere) during the first 420 min, b) between 420 and 1200 min from the start of the reaction. Spectra were recorded every 1 min. c) Plot of absorbance at 476 nm vs. time.

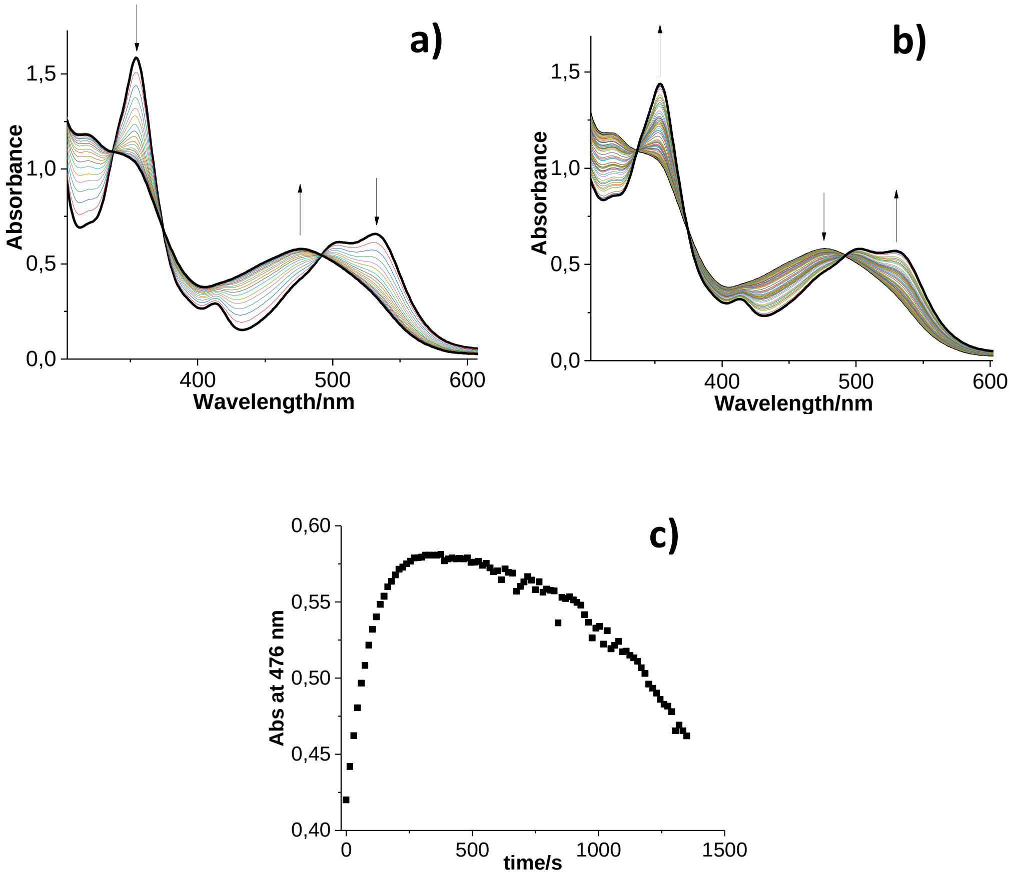

Figure S6. a) Spectral changes observed for the reaction between $CblNO_2$ (8.6×10^{-5} M, obtained by mixing $CblOH_2$ and NO_2^-, $[NO_2^-]/[CblOH_2] = 5$) and $HAsc^-$ (8.6×10^{-4} M) at pH = 5.0 (0.1 M acetate buffer, 25 °C, Ar atmosphere) during the first 400 min, b) between 400 and 1300 min from the start of the reaction. Spectra were recorded every 1 min. c) Plot of absorbance at 476 nm vs. time.
